# Effects of static exercises on hip muscle fatigue and knee wobble assessed by surface electromyography and inertial measurement unit data

**DOI:** 10.1038/s41598-024-61325-7

**Published:** 2024-05-07

**Authors:** Olivia L. Dyer, Mark A. Seeley, Benjamin B. Wheatley

**Affiliations:** 1 Musculoskeletal Institute, Geisinger, Danville, PA USA; 2 Department of Mechanical Engineering, Bucknell University, 1 Dent Drive, Lewisburg, PA 17837 USA

**Keywords:** Gluteus medius, Gluteus maximus, Rectus femoris, Surface electromyography, Inertial measurement unit, Regression, Classification, Biomedical engineering, Orthopaedics

## Abstract

Hip muscle weakness can be a precursor to or a result of lower limb injuries. Assessment of hip muscle strength and muscle motor fatigue in the clinic is important for diagnosing and treating hip-related impairments. Muscle motor fatigue can be assessed with surface electromyography (sEMG), however sEMG requires specialized equipment and training. Inertial measurement units (IMUs) are wearable devices used to measure human motion, yet it remains unclear if they can be used as a low-cost alternative method to measure hip muscle fatigue. The goals of this work were to (1) identify which of five pre-selected exercises most consistently and effectively elicited muscle fatigue in the gluteus maximus, gluteus medius, and rectus femoris muscles and (2) determine the relationship between muscle fatigue using sEMG sensors and knee wobble using an IMU device. This work suggests that a wall sit and single leg knee raise activity fatigue the gluteus medius, gluteus maximus, and rectus femoris muscles most reliably (p < 0.05) and that the gluteus medius and gluteus maximus muscles were fatigued to a greater extent than the rectus femoris (p = 0.031 and p = 0.0023, respectively). Additionally, while acceleration data from a single IMU placed on the knee suggested that more knee wobble may be an indicator of muscle fatigue, this single IMU is not capable of reliably assessing fatigue level. These results suggest the wall sit activity could be used as simple, static exercise to elicit hip muscle fatigue in the clinic, and that assessment of knee wobble in addition to other IMU measures could potentially be used to infer muscle fatigue under controlled conditions. Future work examining the relationship between IMU data, muscle fatigue, and multi-limb dynamics should be explored to develop an accessible, low-cost, fast and standardized method to measure fatiguability of the hip muscles in the clinic.

## Introduction

Following a lower limb injury, physicians commonly assess hip strength to determine if a patient is fit to return to normal activity. One clinically relevant measure of muscle function is muscle motor fatigue, which is a temporary decrease in the ability of a muscle to generate force due to repeated contraction, and differs to other types of fatigue that may be pathological^1,2^. Currently, there are a variety of tests and assessments that measure specific aspects of hip strength and muscle motor fatigue. Two common tests include the HipSIT Test, which has patients perform a clam-shell exercise to measure hip abduction strength, and the Vail Sports Test, which has patients perform a single leg squat, lateral bounding, and jogging to assess hip and core strength^3,4^. However, all of these tests lack direct muscular measurements and require assessment from a trained expert. In addition to the HipSIT test, other methods of strength assessment in the clinic include using a dynamometer, but this tool assumes that the participant is generating a specific amount of force, typically maximum voluntary contraction, which can be inconsistent when assessed across individuals and across a single individual on different days. In addition, this tool is typically used to determine force production during short term contraction, which does not assess muscle fatigue and makes accurate assessment and design of a rehabilitation program a challenge^5–7^. Once muscles begin to fatigue, their recruitment patterns can change, which puts an individual at risk for a musculoskeletal injury^8,9^. A previous review has also shown that while there are various techniques available to assess muscle strength and fatiguability in children, there is no gold standard^10^. Thus, assessing hip muscle fatigue with a low-cost, fast, and simple approach that can be implemented by clinicians remains a current clinical challenge.

Previous work has shown that surface electromyography (sEMG) can be used to characterize muscle motor fatigue by observing a decrease in mean (MNF) frequency of the sEMG signal over time as a static exercise is performed^11,12^. To convert voltage into frequency, a fast Fourier transform is applied to the signal and segmented into time intervals where the MNF can be calculated^13^. Currently, there is no standard method in place to accurately assess and quantify muscle fatigue in the clinic, however the use of sEMG could provide an objective assessment of an individual’s muscle motor fatigue. Previous work has used sEMG to measure muscle fatigue of the lower back muscles while performing variations of the Sorensen test, as well as to characterize muscle fatigue during walking and running, but there is limited information about fatiguability of the gluteus medius, gluteus maximus, and rectus femoris muscles during isometric hip activities^14–18^.

While sEMG can be used to reliably assess muscle fatigue, it requires the use of specialized equipment and training. Inertial measurement units (IMUs) measure translational and rotational accelerations and are commonly used in human motion analysis studies. Assessment of muscle function via vibromyography (vibrational analysis of IMU or accelerometer data) has shown that with increasing muscle fatigue, the vibrational signal amplitude increases and frequency decreases, leading to overall increases in acceleration^19–21^. Accelerometer data from low-cost IMUs that quantify changes in motion at key locations on the body—such as knee acceleration or “wobble” induced by muscle fatigue—could act as a substitute for muscle fatigue estimates from sEMG data. Use of IMUs is advantageous due to their ease of use, portability, lower cost, and they require less expertise than sEMG analysis, making them an ideal device for clinical use^22–25^. For example, Versteyhe et al. used IMU sensors to estimate full knee joint kinematics during various activities and others have implemented IMUs to understand other lower extremity kinematics during walking^22,24,25^. While IMUs are commonly used in kinematic assessments such as understanding joint angles and postural sway, it remains unclear if they can be implemented to understand how knee wobble relates to muscle fatigue^22,24,26^.

Thus, the goals of this study were to (1) identify which specific static activities that can be performed in any clinical setting are well suited to elicit hip muscle fatigue, (2) quantify how these activities affect the fatigue of different hip muscles, and (3) determine if a low-cost, easily accessible IMU placed on the knee could be used as a quantitative substitute for sEMG data to indicate hip muscle fatigue. To achieve these goals, we measured the differences in muscle fatigue of the gluteus maximus, gluteus medius, and rectus femoris with sEMG and differences in knee wobble with IMU data across five commonly performed activities—a single leg squat, wall sit, side leg raise, hip extension, and knee raise. We hypothesized that (1) there would be a decrease in the mean sEMG frequency, indicative of muscle fatigue, and an increase in acceleration, indicative of knee wobble, as the duration of the activity increased and that (2) there would be a correlation between mean frequency (muscle fatigue) and measures of knee acceleration (knee wobble).

## Materials and methods

**Figure 1:**
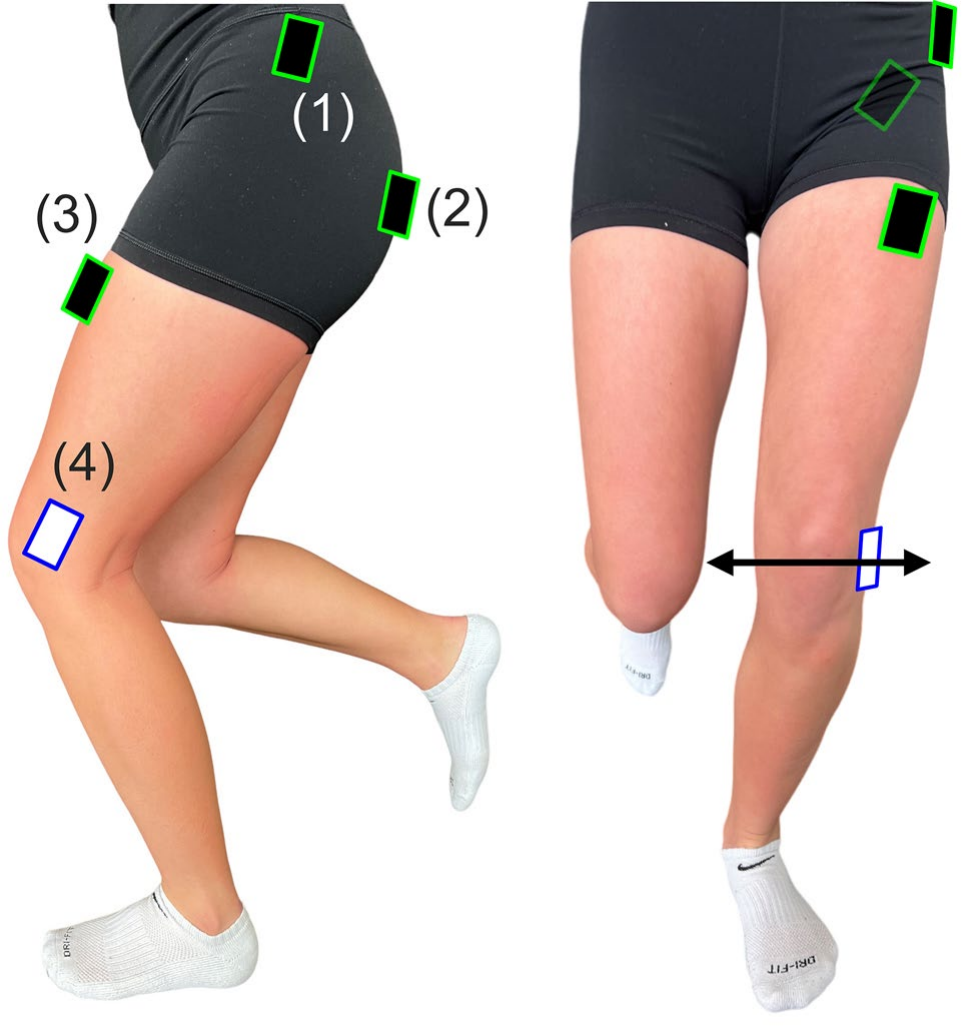
Sensor overview. Three surface electromyography (sEMG) sensors (black and green boxes) placed on the (1) gluteus medius, (2) gluteus maximus, and (3) rectus femoris muscles. An (4) inertial measurement unit (IMU) sensor (white and blue boxes) was placed on the lateral portion of the knee. Knee wobble was defined as resultant translational acceleration of the three-axis IMU and is represented in the black arrow.

Twenty-four participants (14F, 10M) with ages ranging from 18–21, were recruited to participate in a fatigue analysis study. Participants were identified as healthy individuals with no current or chronic musculoskeletal injuries or neuromuscular diseases, with a target of at least n = 20 for our sample size based on prior work^11,16,19,27,28^. This study was approved by the Bucknell University IRB (number 2324), all research was performed in accordance with relevant guidelines, and informed consent was obtained from all subjects prior to data collection. Following informed consent, three sEMG sensors (Delsys Tringo Avanti Sensor, Natick, MA, used in surface electromyography mode) were placed on the gluteus medius, gluteus maximus, and the rectus femoris muscles, respectively of either the left or right knee (Fig. 1). Additionally, an inertial measurement unit (Delsys Tringo Avanti Sensor, Natick, MA, used in translational acceleration model) was placed on the lateral portion of the knee (Fig. 1) that recorded three-axis translational accelerations, from which we calculated the resultant acceleration. The knee was chosen as the location on the body most likely to be subject to the largest translations and accelerations during static activities such as a squat or wall sit, assuming the feet are fixed and the trunk (center of mass) is subject to minimal movement. Participants were allowed to self-select their tested limb, and if they had no preference a random limb was chosen. If one leg had previous history of an acute musculoskeletal injury, the other leg was chosen for testing. To ensure proper sensor adhesion, hair was shaved as necessary, and all skin was cleaned with alcohol and an adhesive spray was used on each sensor location^29^. Data collection occurred at a sampling frequency of 4370 Hz and 370 Hz for the sEMG sensors and IMU, respectively^30,31^.

**Figure 2:**
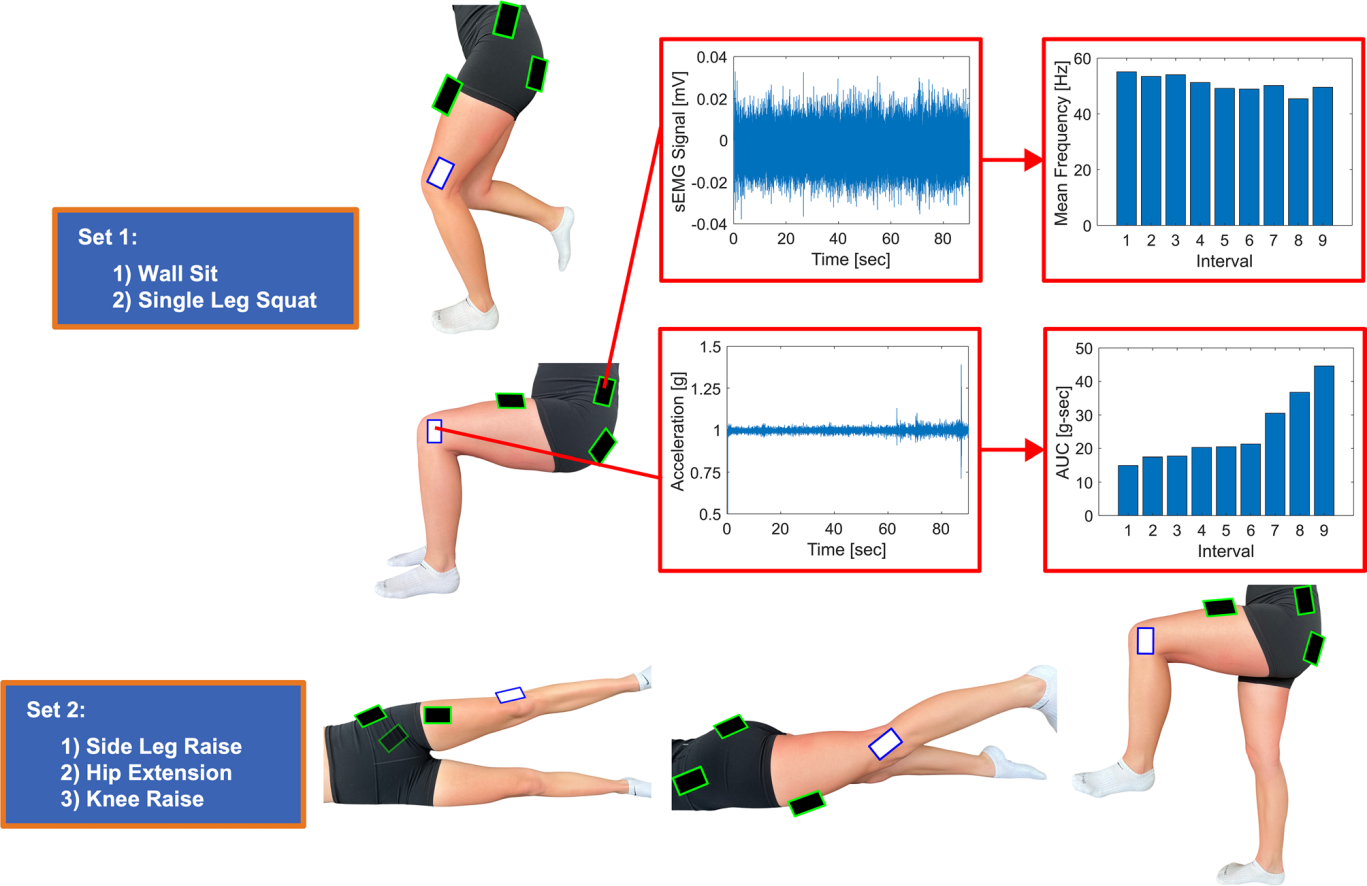
Schematic of experimental overview. (**A**) Two sets of activities were performed, the primary set included a wall sit and a single leg squat, the secondary set included a side leg raise, hip extension, and knee raise. The activities within each set were randomized, but the primary set of exercises always preceded the secondary set of exercises. (**B**) Raw sEMG and resultant translational acceleration data was recorded and was analyzed to determine the mean frequencies, absolute electrical signal, and acceleration measures within each interval for each activity.

A literature search was conducted to determine commonly performed exercises to measure hip strength in the clinic, and a list of the most common static exercises were selected for this study: wall sit, single leg squat, lateral recumbent side leg raise (hip abduction), prone hip extension, and standing single knee raise (standing hip flexion)^3,4,6,7,32^. Participants performed five static exercises for 90 s each, grouped into two sets, the primary set (most likely to be performed clinically) included a wall sit and single leg squat and the secondary set (for isolating each muscle) consisted of a side leg raise, hip extension, and knee raise (Fig. 2). Each participant was instructed to maintain the specific position (wall sit, hip abduction, etc.) for a total of 90 s (static exercise) and was provided verbal encouragement throughout the exercise to maintain their appropriate form. For the single leg squat and knee raise exercise, participants were allowed to place one hand on a wall to maintain balance, but were instructed not to bear weight on the wall. The exercises within each set were randomized, and the primary set always preceded the secondary set of exercises to ensure subjects were not fatigued for these primary exercises.

All data analysis was performed in MATLAB and left–right limb data were grouped together. The sEMG data was filtered with an eighth order Butterworth filter with a lower and higher cutoff frequency of 20 Hz and 400 Hz, respectively^27^. The first two seconds and last two seconds of each static exercise were removed to prevent artifacts from the beginning and end of each exercise and the remaining 86 s was separated into ten intervals. The filtered and segmented data was then analyzed using a fast Fourier transform (FFT) to determine the mean frequency (MNF) for each of the nine intervals^11,33^. We used the *fft* function in MATLAB with no windowing, a total FFT length of 37,582 data points (8.6 s at 37,582 Hz), and no overlap between segments. The mean of the absolute electrical signal, which is a nonlinear correlate to muscle force under isometric conditions and can be interpreted as an approximation of muscular effort or contraction level, was also determined for each interval^28,34–36^. The acceleration data was filtered using a 4th order low pass Butterworth filter with a cutoff frequency of 10 Hz based on prior research that used cutoff frequencies ranging between 6–20 Hz for accelerometer data, in particular the use of 10 Hz to filter data without the loss of peak accelerations^37,38^. After the resultant acceleration was calculated, and the data was segmented into the same ten intervals as the sEMG data. The maximum sensor acceleration within each interval, area under the absolute value of the resultant acceleration curve (AUC), sum of squared acceleration values, and zero cross rate (the average amount of time between signal crosses of the x-axis or zero) for each interval were calculated. These four measures were interpreted as characterizations of knee wobble. All MNF, absolute sEMG signal, and accelerometer data were then normalized to the inter-trial maximum. Muscle motor fatigue was identified as a decrease in MNF over the duration of the activity or a negative regression slope, while an increase in knee wobble was defined as an increase in acceleration over the duration of the activity or a positive regression slope^13^. An increase in absolute sEMG signal (or no change to the signal) throughout an activity signified that the muscle effort requirements to maintain the static pose did not decrease throughout the activity. Specifically, decreases in MNF paired with increases in (or no change to) the absolute signal are indicative of muscle fatigue^28^.

For statistical analysis, paired two-way *t*-tests were conducted comparing MNF for the first and last intervals for all three muscles and all four acceleration measures. Linear regressions were performed on all MNF data and MNF data subdivided into specific activities and specific muscles to determine the R^2^ and slope for each exercise and each muscle. Similar linear regressions were performed on all absolute sEMG signal data and acceleration data. One-way analysis of variance (ANOVA) tests were performed on regression slope coefficients to compare fatigue and knee wobble across muscles and activities^16^. Multivariate and stepwise linear regressions were performed on all four accelerometer measures against the sum of MNF from all three muscles to determine the extent to which muscle fatigue could be identified from sEMG data.

To more carefully investigate potential differences in muscle fatigue across activities, we identified a subset of the data that included only individual “responders” to each activity. We classified each set of data as a “responder” to an activity as follows: a decrease in sEMG frequency, indicating fatigue, must have a negative regression slope and a regression p-value less than 0.05, while an increase in acceleration measures, indicating increased knee wobble, must have a stepwise linear regression p-value less than 0.05. Knee wobble responder results were used as predictors of at least one muscle fatigue responder. A K-nearest neighbors classification algorithm was also used to predict muscle fatigue response based on all acceleration data. Briefly, this model used a 27 neighbors with a Chebyshev distance metric, a Kruskal Wallis feature selection, modified misclassification costs (weights of 1 for true positive/predicted negative and 4 for true negative/predicted positive), and five-fold model validation^39,40^. All statistical analysis was performed in MATLAB with significance set at α = 0.05 for *t*-tests and ANOVAs.

## Results

**Figure 3:**
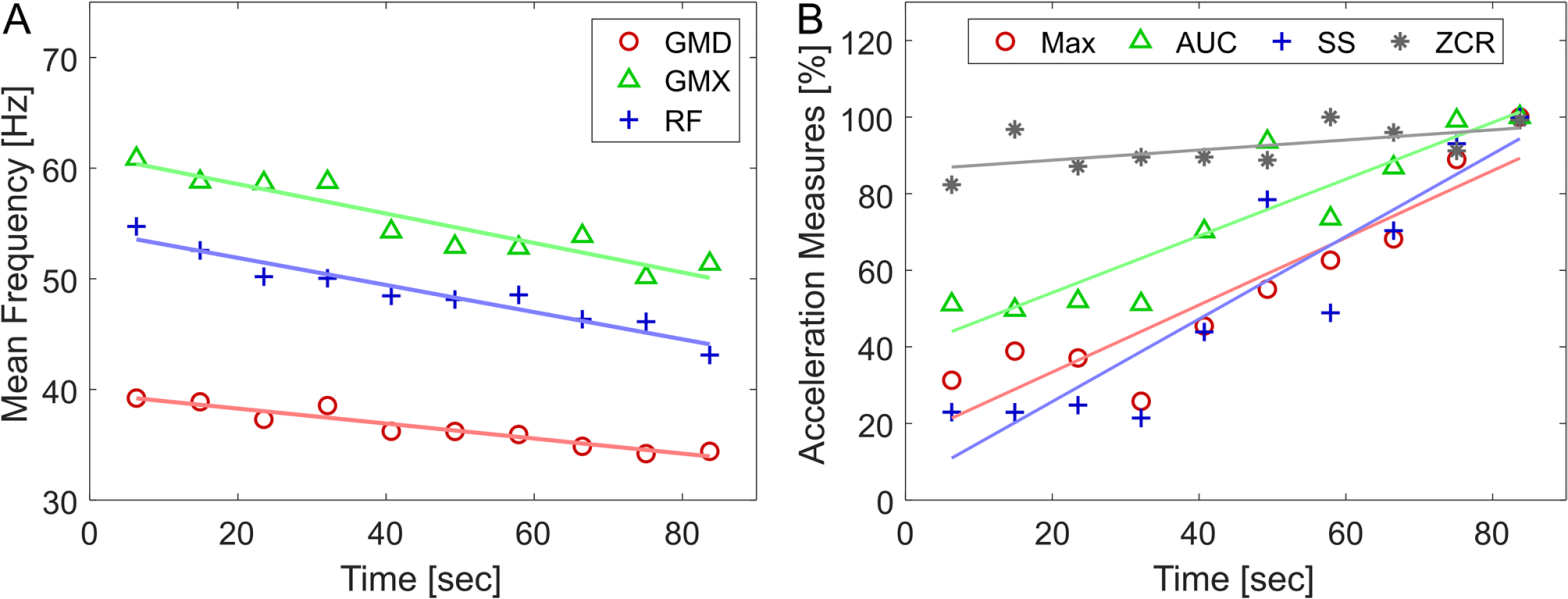
Representative data from subject 21 during the knee raise activity, showing data from the ten intervals and linear regression fits. (**A**) Muscle sEMG mean fatigue (MNF) for the gluteus medius (GMD—red circles), gluteus maximus (GMX—green triangles), and rectus femoris (RF—blue crosses). (**B**) Acceleration data, showing the maximum acceleration (max—red circles), area under the absolute acceleration curve (AUC—green triangles), sum of squares (SS—blue crosses), and zero cross rate (ZCR—gray stars).

Linear regressions for all normalized MNF showed a statistically significant negative effect of time (a decrease in MNF) for all three muscles (p < 0.001, representative results shown in Fig. 3). For all inertial measurement unit data, linear regressions showed a statistically significant positive effect of time (an increase in knee wobble) for maximum acceleration, resultant acceleration area under the curve, and acceleration sum of squares (p < 0.001, representative results shown in Fig. 3), and a negative effect of time for normalized zero cross rate (p = 0.022). Total electrical activity quantified by the mean of the absolute sEMG electrical signal increased for all three muscles (p < 0.001), and regression slopes broken out by muscle and activity were either positive (increased muscle effort, p < 0.05) or not statistically significant (no change in muscle effort, p > 0.05) (Supplementary Table S3).

**Figure 4:**
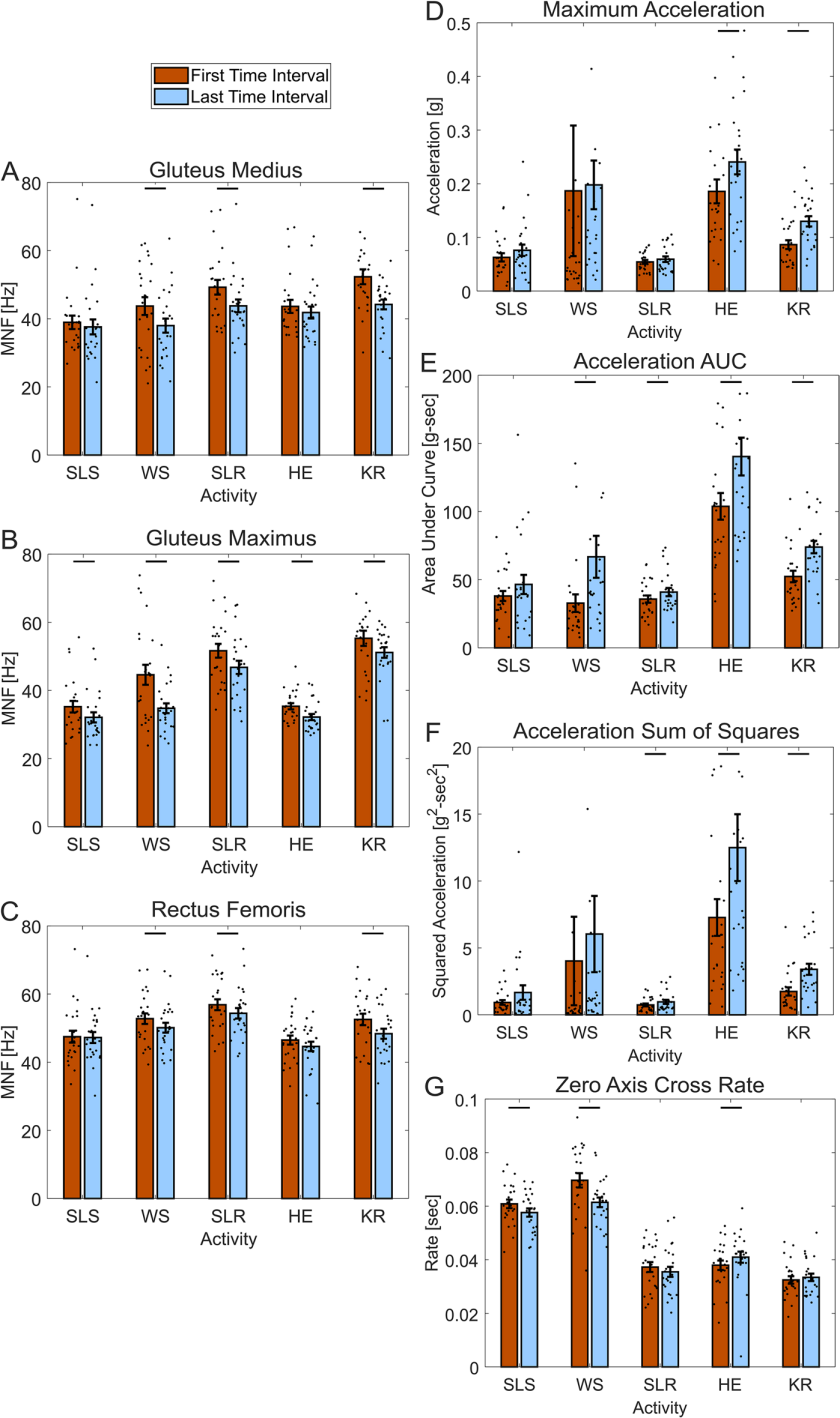
Bar graphs of the first last intervals of sEMG and acceleration data, showing differences across different activities. Mean frequency (MNF) for each activity is provided for the (**A**) gluteus medius muscle, (**B**) gluteus maximus muscle, and (**C**) rectus femoris muscle. Mean accelerometer data for each activity of the (**D**) maximum acceleration, (**E**) area under the rectified acceleration curve, (**F**) sum of squared acceleration, and (**G**) acceleration zero-axis cross rate. All graphs show average with standard error bars, with p < 0.05 for statistical comparisons within each activity denoted by horizontal lines between bars.

On average, the mean frequencies (MNF) decreased for each muscle from the first time interval to the last time interval of each activity (Fig. 4A–C), however this was not statistically significant for all activities and all muscles. For the gluteus medius muscle, there was a statistically significant decrease for the wall sit (p = 0.0018), side leg raise (p < 0.001), and knee raise activities (p < 0.001) (Fig. 4A). For the gluteus maximus, there was a statistically significant decrease for all of the activities—single leg squat (p = 0.017), wall sit (p = 0.0011), side leg raise (p < 0.001), hip extension (p < 0.001), and knee raise (p = 0.025) (Fig. 4B). For the rectus femoris, the wall sit (p = 0.021), side leg raise (p = 0.013), and knee raise (p < 0.001) activity had a statistically significant difference in mean frequency for the first and last intervals (Fig. 4C).

On average, the maximum acceleration, resultant acceleration area under the curve, and acceleration sum of squares increased from the first interval to the last interval of each activity, which was not the case for the acceleration zero cross rate (Fig. 4D–G). For the maximum acceleration, this increase was significant for the hip extension (p = 0.047) and knee raise activity (p < 0.001) (Fig. 4D). For the acceleration area under the curve (AUC), this increase was significant for the wall sit (p = 0.012), side leg raise (p = 0.015), hip extension (p = 0.0016), and knee raise activity (p < 0.001) (Fig. 4E). For the sum of squared acceleration area, this increase was significant for the side leg raise (p = 0.024), hip extension (p = 0.016), and knee raise activity (p < 0.001) (Fig. 4F). For the acceleration zero cross rate, there was a statistically significant decrease for the single leg squat (p = 0.015), wall sit (p < 0.001), and an increase for the hip extension (p = 0.038) (Fig. 4G).

**Figure 5:**
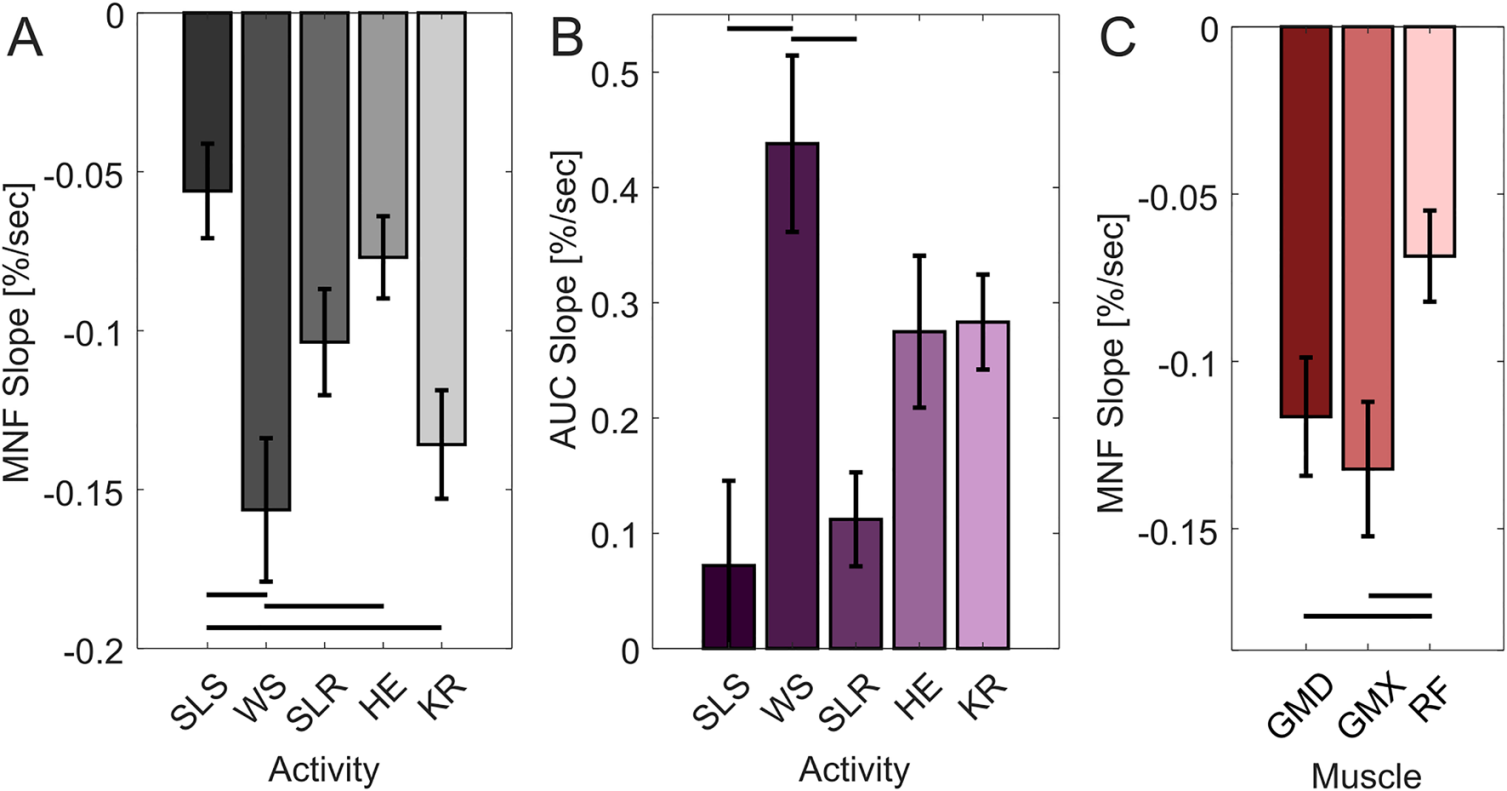
All univariate linear regression results showing differences in sEMG data and acceleration data across different activities and different muscles. (**A**) Mean frequency (MNF) slopes by activity, (**B**) inertial measurement unit area under the resultant the rectified acceleration curve (AUC) slopes by activity, and (**C**) mean frequency slopes by muscle. *SLS* single leg squat, *WS* wall sit, *SLR* single leg raise, *HE* hip extension, *KR* knee raise, *GMD* gluteus medius, *GMX* gluteus maximus, *RF* rectus femoris. All graphs show average with standard error bars, with p < 0.05 denoted by horizontal lines between bars.

Normalized univariate linear regression results also reveal muscle and activity specific changes in muscle fatigue, electrical signal, and knee wobble (Fig. 5, Supplementary Figs. S1–S3 and Tables S1–S3). The average slopes for all muscles across all activities were negative, indicating muscle fatigue during all five activities, with rectus femoris MNF as the only non-statistically significant result at p = 0.096 (Supplementary Fig. S1 and Table S1). Comparing between activities, the wall sit slope was more negative than the single leg squat (p < 0.001) and the hip extension (p = 0.0088) activities, and the knee raise slope was more negative than the single leg squat (p = 0.0092). Comparing between activities for knee accelerometer area under the curve AUC, the wall sit slope was greater than the single leg squat (p < 0.001) and side leg raise (p = 0.0026) activities, indicating greater knee wobble. Comparing between muscles, the gluteus medius and gluteus maximus slopes were more negative than the rectus femoris (p = 0.031 and p = 0.0023, respectively), which indicates more muscle fatigue during the activities.

**Figure 6:**
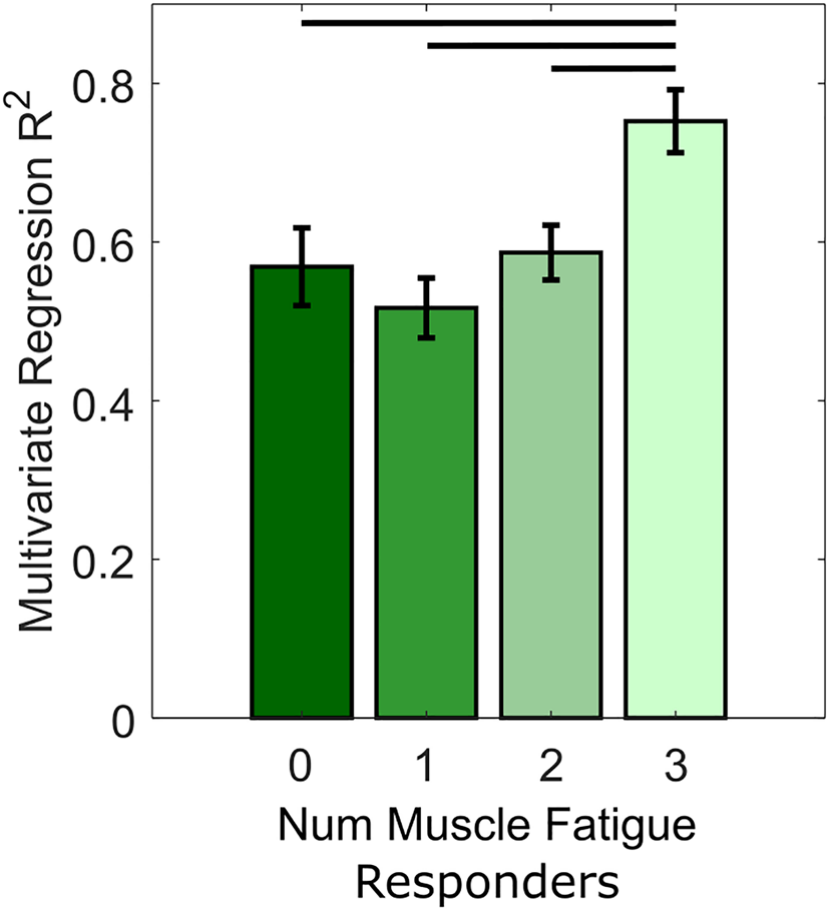
The R^2^ values determined from multivariate linear regressions of all four acceleration measures (maximum acceleration, area under the resultant acceleration curve, sum of squares, and zero cross rate) as input variables with the sum of the MNFs as the response. Regression results are shown for 120 trials, which incorporates all acceleration data and all muscle MNF data for each trial. All graphs show average with standard error bars, with p < 0.05 denoted by horizontal lines between bars.

Regarding muscle fatigue responder (if a negative regression slope and p < 0.05), of the 120 total trials (24 subjects, 5 trials per subject), 60 were classified as gluteus medius responders, 72 were classified as gluteus maximus responders, and 50 were classified as rectus femoris responders. Multivariate linear regressions results for all 120 trials, which used all four acceleration measures as input variables and the sum of MNF across all three muscles as the response, show that with more muscle responders (greater fatigue) the regression is able to explain a greater amount of variance (Fig. 6). Specifically, with all three of the four muscles classified as responders (n = 19/120), the R^2^ of the multivariate regression was greater than when there were zero responders (n = 20/120, p = 0.0497), one responder (n = 37/120, p = 0.0013), or two responders (n = 44/120, p = 0.035). Overall, the multivariate regression explained a moderate amount of the total variance in MNF (average R^2^ ranged from 0.52 to 0.75). Stepwise linear regression classified 102 of the 120 total trials as knee wobble responders (effect of time on against one or more accelerometer measures p < 0.05).

**Figure 7:**
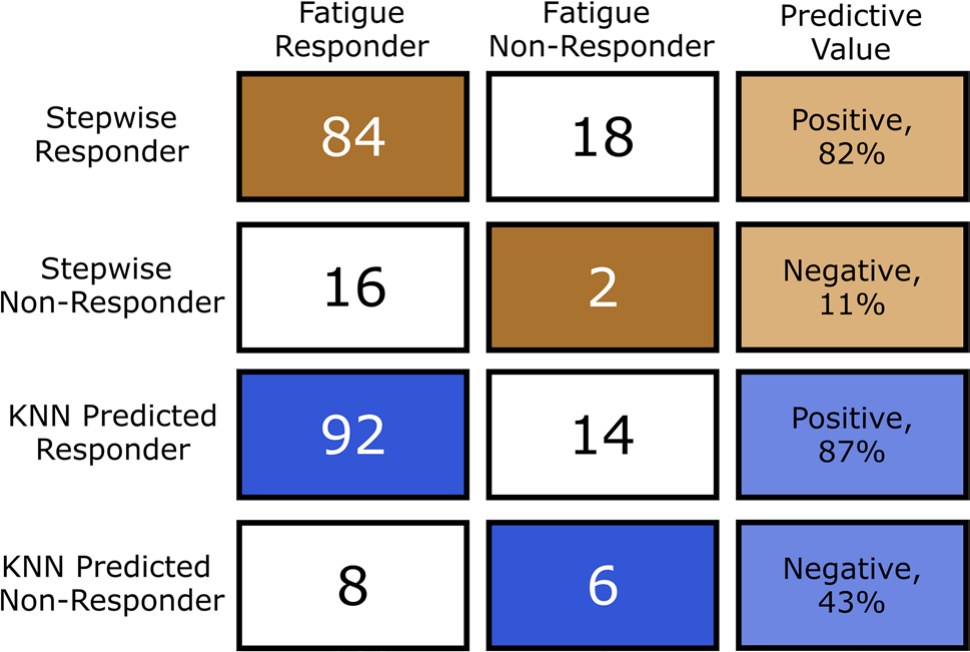
Knee wobble as a predictor of muscle fatigue. A stepwise linear regression responder (effect of time on accelerometer data significant at α = 0.05) had an 82% positive predictive value (84/102) for the fatigue of one or more muscles within an activity and an 11% negative predictive value (2/18) for no fatigue of all three muscles within an activity. A K-nearest neighbors classification algorithm had an 87% positive predictive value (92/106) for the fatigue of one or more muscles within an activity and a 43% negative predictive value (6/14) for no fatigue of all three muscles within an activity.

Stepwise linear regression knee wobble responder results were found to have an 82% positive predictor value of a trial in which one or more muscles were also fatigue responders, while the negative predictive value was only 11% (Fig. 7). The K-nearest neighbor classification yielded improved classification results compared to the stepwise regression, with an 87% positive predictive value and a 43% negative predictive value (Fig. 7).

## Discussion

This work presents a hip muscle fatigue analysis study with 24 participants who all performed a single leg squat, wall sit, side leg raise, hip extension, and knee raise activity for 90 s, with the goal of assessing how different hip muscles were fatigued by these activities. Three surface electromyography (sEMG) sensors were placed on the gluteus medius, gluteus maximus, and rectus femoris muscles, and an inertial measurement unit (IMU) was placed on the lateral portion of the knee. There was, on average, a decrease in mean frequency (MNF) for each muscle within each activity, indicating muscle fatigue, and an increase in acceleration, indicating more knee wobble as the duration of the activity increased, thus supporting our hypotheses that these five activities would elicit muscle fatigue and knee wobble. The wall sit elicited the most muscle fatigue and greatest knee wobble across all activities, which suggests it may be well suited for clinical use. We also found that a K-nearest neighbors classification algorithm was able to use acceleration data to predict muscle fatigue with an 87% positive predictive value, which provides some support for our hypothesis on IMU efficacy for predicting muscle fatigue. However, a 43% negative predictive value suggests further work is required to reliably and consistently predict muscle fatigue and/or muscle weakness with IMU data. To the best of the authors’ knowledge, this study was the first to explore the relationship between muscle fatigue assessed with sEMG and lower extremity wobble assessed with an IMU during a static exercise.

This work was not without limitations. We did not incorporate a limb-specific analysis to measure imbalances prior to the experiment or during the experiment for each participant since we only analyzed one limb, however expanding our approach to include both limbs could be employed in future work. While sEMG is commonly used to measure muscle activity and estimate characteristics such as muscle fatigue and activation timing, the optimal procedure for analyzing the raw signal to estimate muscle fatigue remains unclear, as short-term frequency analysis cannot reliably predict muscle fatigue^11,13,15,27,41–43^. In addition, sensor preparation, including sensor placement, adhesion of the sensors to the skin, and the location of the sensor relative to the active area of muscle contraction are crucial to obtain quality signals from the muscles of interest, and may vary between participants^12^. Thickness of the skin, adipose tissue, and the bipennate nature of the rectus femoris can also affect the quality of the electrical signal across individuals^12,44^. IMU devices, while cheap and easily accessible, also rely on consistent sensor placement, and can have poor velocity and displacement estimations due to integration errors, although we did not use velocity or displacement estimations in this work^22,23,45^. To reduce fatigue from performing too many exercises, only five activities were selected—two “general” activities (the single leg squat and wall sit) and three “targeted” activities (the side leg raise for the gluteus medius, hip extension for the gluteus maximus, and knee raise for the rectus femoris). We also used only one IMU sensor on the lateral knee for simplicity (potential integration into clinical use) and consistency across exercises. Finally, only healthy participants were used in this study, so it is unclear if injured participants would elicit a different muscle fatigue response or follow a similar trend that was observed in the healthy group.

One challenge of this work was the comparison of muscle fatigue as assessed by sEMG data across different activities that have different biomechanical demands. The challenge of assessing and comparing muscle fatigue across different activities and different individuals is due to the broad definition of fatigue as a biophysical phenomenon and difficulties determining biomechanical measures such as muscle force^11,46–49^. For example, comparisons of muscle force requirements to maintain a wall sit versus a prone hip extension would require highly invasive force transducer surgery, estimations conducted via biomechanical analysis that would include motion capture data or static alignment measurements, force data, and subject-specific musculoskeletal modeling, or other approaches such as experimental and computational analyses of intramuscular pressure^50–55^. Therefore, this work aims to compare these static exercises through the context of hip muscle fatigue as assessed by sEMG for use as easy to implement clinical tests. Although each of the static exercises used in this study incorporated hip flexion or extension, some exercises incorporated knee flexion (wall sit, single leg squat, and single leg raise) and the wall sit involved contact with both the floor and the ground. For example, we did not seek to determine how these exercises may best be used in a rehabilitation program. Additionally, some exercises such as the single leg squat may be more prone to knee wobble induced from instability and not muscle fatigue, which further supports the notion of the wall sit as a reliable exercise to assess muscle fatigue. Future work to incorporate a more detailed biomechanical analysis (such as incorporating motion capture data and force measurements) could provide insight into different muscle force demands across activities, although such an experimental setup would not be reasonable for fast, easy, and consistent clinical use.

The average slope of the sEMG mean frequency data was negative for all muscles and all activities (Fig. 5), suggesting that each activity is able to elicit at least some muscle fatigue in the gluteus medius, gluteus maximus, and rectus femoris muscles. Furthermore, our findings of either no change to or increases to the mean absolute sEMG electrical signal (an approximation of muscle effort) paired with decreases in MNF further support the notion of muscle fatigue, as characterized by the “JASA” method by Luttmann et al.^11,28^. For example, decreases in the signal could indicate decreasing muscle force demands—such as reducing squat depth during the single leg squat—which could confound our MNF measures. Thus, we are confident that the static activities used in this study and our specific experimental approach elicited muscle fatigue of the gluteus medius, gluteus maximus, and rectus femoris. Comparing across activities, the wall sit elicited more muscle fatigue than the single leg squat and hip extension (p < 0.001 and p = 0.0088, respectively, Fig. 5A) and the knee raise elicited more muscle fatigue than the single leg squat (p = 0.0092, Fig. 5A) as assessed by the slope of the MNF regression curves. While there is no universally accepted clinically relevant decrease in MNF to compare across muscles and activities, the wall sit MNF slope was nearly three times that of the single leg squat slope (Fig. 5), and was more than two times greater for each muscle (Fig. S1 and Table S1). Thus, along with the first/last interval comparison results (Fig. 4) and the increased acceleration area under the curve (AUC) slope for the wall sit compared to the single leg squat and side leg raise (p < 0.001 and p = 0.0026, respectively, Fig. 5B), we suggest that the wall sit may be easily performed in the clinic to most reliably elicit muscle fatigue and knee wobble and is a viable candidate for further study. The lack of response for the single leg squat could be because as the exercise is performed, individuals compensate for the muscle fatigue and drift upwards, thus reducing the force requirements by each muscle and thus delaying fatigue. This stance drifting is not as possible with the wall sit activity.

Comparing MNF between the first and last intervals for each muscle and each activity, the gluteus maximus MNF decreased for all activities (p < 0.05, Fig. 4B), and the average gluteus slopes were more negative than the average rectus femoris slope (Fig. 5C), suggesting fatigue of the glute muscles could be most reliably measured with sEMG. Each targeted activity produced a statistically significant decrease in MNF—the gluteus medius for the side leg raise, the gluteus maximus for the hip extension, and the rectus femoris for the single leg raise (Fig. 4, p < 0.05), supporting our experimental design.

Prior studies have used a linear regression to determine the decrease in mean or median frequency of an sEMG signal during a static or dynamic activity, indicating muscle fatigue^13,15,16,56–58^. Some studies, including ours, have the participant hold an isometric contraction for 60 to 120 s followed by 2–10 min of rest before another activity is performed, whereas others have participants hold a position until failure^42,56–59^. The use of a fast Fourier transformation to calculate the mean frequency over a time interval is commonly used in fatigue analysis studies, but other methods, such as observing the amount of times the raw sEMG signal crosses the x-axis, counting the number of peaks, and characterizing changes in the sEMG amplitude from its root-mean-square values have been proposed and could be used in future work^11,60^. Our assessment of increases in knee wobble as a result of increasing muscle fatigue is based on prior work that has shown correlations between sEMG data and vibromyography data during fatigue and voluntary contraction studies^19,20^.

One study by Coorevits et al. found an approximate negative sEMG frequency slope of − 0.2%/s for the gluteus medius muscle while performing the Biering-Sorensen test^16^. Champagne et al. similarly observed an average negative median sEMG frequency slope of − 0.15%/s for the gluteus medius muscle during variations of the Sorensen test^15^. For the gluteus maximus, another study by Coorevits et al. showed fatigue during the Biering-Sorensen test at an average median sEMG frequency slope of − 0.15%/s^56^. A fourth study found a mean sEMG frequency slope of approximately − 0.3%/s for the rectus femoris muscle while performing an isomeric knee extension exercise^58^. Our average MNF slopes range from approximately − 0.5%/s for the single leg squat to approximately − 0.15%/s for the wall sit (Fig. 5A). Thus, our fatigue results show a reasonable agreement with previously published work, giving confidence in our muscle fatigue findings.

**Figure 8:**
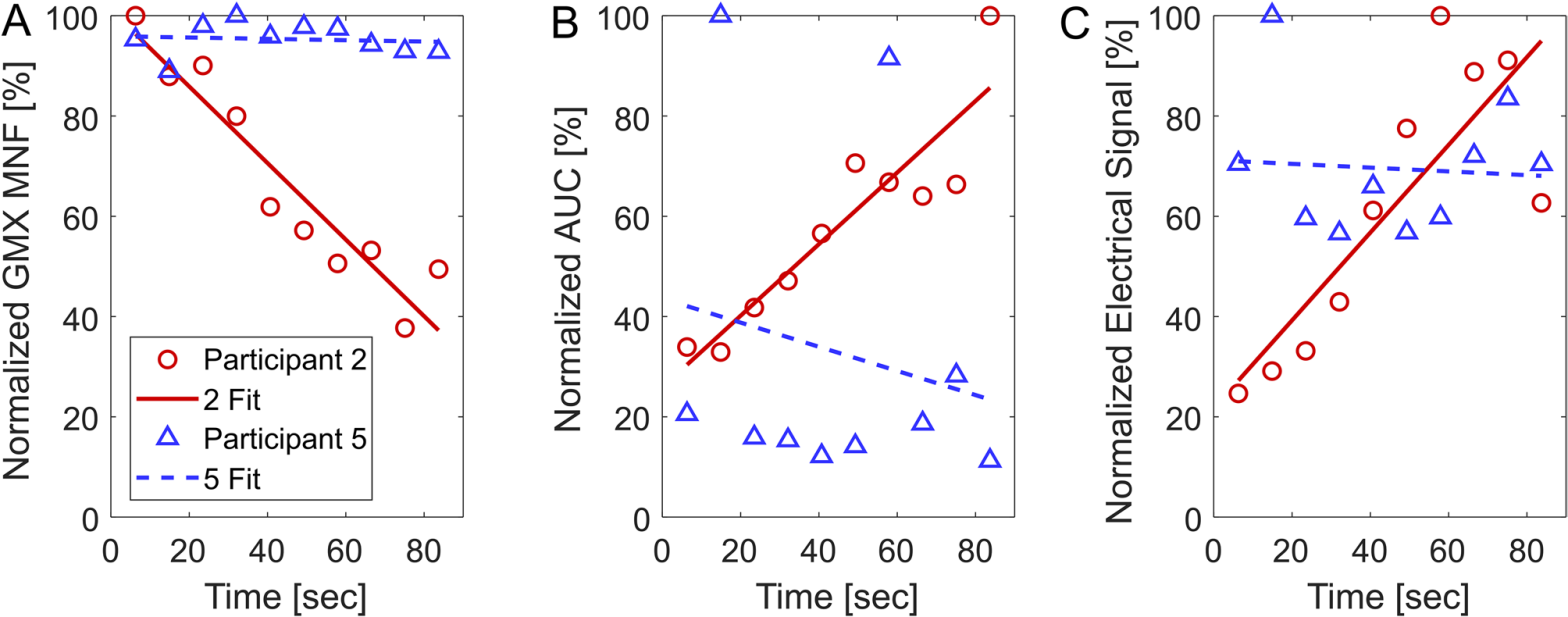
Representative comparison of a fatigue response (“responder”) and a non-responder for the wall sit activity showing (**A**) the normalized gluteus maximus mean frequency (MNF), (**B**) the normalized area under the translational acceleration curve (AUC), and (**C**) the normalized electrical signal. Participant 2 (red circles) shows a clear decrease in MNF, increase in AUC, and increase in electrical activity over the nine intervals, while participant 5 (blue triangles) was defined as a non-responder and had no clear changes in these measures over the nine intervals.

To further investigate the variability between subjects and reliability of exercises, we generated a subset of activities classed as “responders” (Fig. 8). A negative slope for MNF regression coupled with a p-value < 0.05 was considered a muscle fatigue responder to the activity, and an acceleration data stepwise linear regression with a p-value < 0.05 was considered a knee wobble responder. Thus, each activity has four possible responders—three muscle fatigue MNF regressions and one knee wobble AUC regression. Analyzing this data set gave us greater insight into the effectiveness of IMU data to predict muscle fatigue, as responders can be classified in binary fashion. While the mechanisms for responders versus non-responders remains unclear, it is likely due to a combination of different fatigue resistance among individuals, possible changes to contraction level or effort throughout the exercise, or other experimental artifacts such as poor static exercise form^28^. For example, participant two during the wall sit activity was classified as a muscle responder for the gluteus maximus, and also showed an increase in acceleration AUC and an increase in electrical signal, while participant five did now show changes in these values throughout the activity (Fig. 8). Further investigation into these exact mechanisms in future work would be a benefit for clinical application.

Multivariate regressions were performed on acceleration data for each interval as the input variables (the maximum resultant acceleration, area under the rectified acceleration curve, sum of squares, and acceleration zero cross rate) and mean frequency data (summed across all three muscles) as the response. The goal of these regressions was to determine the extent to which knee wobble was correlated to muscle fatigue. Regression results showed that the amount of MNF variance explained by acceleration data increased with the number of muscle responders, as the R^2^ of the regression when all three muscles classified as responders was greater than zero, one, or two responders (p < 0.005, Fig. 6). This finding shows that with greater muscle fatigue, specifically fatigue of more muscles, or a lower endurance tolerance for some individuals, knee wobble became more pronounced, which suggests that knee wobble could be more reliably used to estimate extreme fatigue or impairment. While the amount of explained variance was moderate (mean R^2^ = 0.55–0.75), this multivariate linear regression approach was not able to reliably correlate muscle fatigue and knee wobble for individual activities. This result is unsurprising as hip strength and knee instability are dependent on multiple muscles, which may fatigue at different rates and to different levels across activities and individuals^61^.

However, the responder subset data analysis revealed promise for the use of IMU data as a binary indicator of muscle fatigue. Specifically, knee wobble responders had an 82% positive predictive value for at least one muscle fatigue responder (Fig. 7), although this came with a poor negative predictive value of 11% due to the high number and overlap of both muscle fatigue responders and knee wobble responders. This finding may be partially because one IMU is used to predict whether or not fatigue could occur in three muscles. A K-nearest neighbors classification algorithm, which used a supervised learning approach, yielded improved classification results with an 87% positive predictive value and a 43% negative predictive value. This classification method used all four acceleration measures (maximum, area under the curve, sum of squares, and zero cross rate) across all ten intervals as input variables to predict either no fatigue or fatigue of one or more muscles.

While a negative predictive value of 43% would make direct clinical implementation of this exact method a challenge, the use of only the resultant acceleration data from a single IMU shows good promise for future efforts to link lower extremity wobble and muscle fatigue during static exercise, particularly for hip muscles. Unfortunately, this approach was also not effective at predicting individual hip muscle fatigue, which may be best suited for targeted sEMG analysis. This work suggests one or more simple, low-cost IMUs with moderate data analysis could potentially provide insight into hip muscle motor fatigue in a clinical setting, particularly as a screening tool in addition to other clinical measures.

Each exercise took 90 s to complete and required sensors being placed on the participant and minimal space to perform the activities, suggesting such a test would be easily performed in the clinic. Wahl et al. found a wall sit time-to-failure of approximately 140 s in a highly trained cohort (while our cohort was simply young healthy volunteers), thus expanding the time of the wall sit test in the clinic could be easily implemented^62^. Comparatively, the VAIL Sports test requires a larger space to perform all of the exercises, which is a challenge to perform in the clinic^4^. Additionally, the VAIL sports test can take close to 20 min and requires subjective assessment from a clinician, a considerable increase in time as compared to a 90 s test and required expertise as compared to a packaged sensor that may be able to collect and analyze data in real time. An advantage of the VAIL sports test is it requires dynamic movements and is commonly used as an assessment following ACL reconstruction, but the lack of quantitative data and the subjective assessment causes some difficulty tracking progress longitudinally. The HipSIT test uses a dynamometer to assess hip muscle strength during side lying activities, which means it can be completed in the clinic and captures quantifiable data with a history of reliability^3^. The HipSIT test is able to provide information about the collective strength of the hip stabilizer muscles, but is not muscle specific (similar to our knee wobble results), is not designed specifically to assess fatiguability, and requires training and implementation of a dynamometer. In conclusion, each method has strengths and drawback, and there is consensus that a lack of a golden standard test to assess hip strength makes it difficult to determine which test is the most ideal^3,4^.

Future work could include additional IMUs to increase the predictive power of accelerometer data in determining hip muscle fatigue in an easy, low-cost, and quantitative approach. Previous work has shown that IMUs can characterize lower extremity joint kinematics with reasonable accuracy^24,25,63,64^ and identify fatigued versus non-fatigued gait profiles^65,66^, thus the use of IMUs to identify hip fatigue during static exercises is worth future study. Previous work has shown that during fatigue-inducing contractions of the lumbar extensor muscles, the posture and ankle flexion of individuals changed, so additional IMU sensor placement on the torso and ankle may be able to better capture how the entire body changes in response to muscle fatigue, especially for standing activities^26^. Additional studies have observed as muscle fatigue occurs of the lower limbs, different core muscle recruitment strategies are utilized by the body resulting in changes in torso sway and stability, further suggesting that additional IMU sensor placement may provide more insight into a predictive relationship between accelerometer data and sEMG frequency fatigue data^67,68^. Expanding the cohort of participants to include injured return to sport individuals and expanding methods to assess perceived exertion from each exercise could also help to strengthen our understanding of which exercises are able to best fatigue the hip muscles. Lastly, different methods of data analysis (more advanced machine learning, nonlinear data analysis, etc.) could help to elucidate patterns and trends in the data to find stronger relationships between IMU accelerometer data and the sEMG frequency data.

## Conclusion

A muscle motor fatigue analysis was performed to determine which activities among a single leg squat, wall sit, side leg raise, hip extension, and knee raise fatigued the gluteus medius, glutes maximus and rectus femoris muscles most effectively and how knee wobble changed throughout these activities, as characterized by acceleration data acquired by an inertial measurement unit. Muscle fatigue was observed in all activities and was indicated by a negative average slope of the mean surface electromyography frequency over the duration of the activity. A positive slope for the area under the acceleration curve over the duration of the activity suggests that as muscle fatigue occurred, knee wobbled increased. It was determined that the wall sit activity most reliably fatigued the three muscles, as evaluated by comparing linear regression results for muscle fatigue and knee wobble. It was also found that knee wobble could be used as an indicator of fatigue, but more work is needed in this area to strengthen the relationship between IMU data and muscle fatigue. In the future, development of an accessible, low-cost, fast, and standardized method to measure fatiguability of the hip muscles will allow for clinicians to quantitatively assess if a person can resume normal levels of activities following a lower limb injury.

### Supplementary Information

Supplementary Information.

## Data Availability

The datasets generated during and/or analyzed during the current study are available from the corresponding author on reasonable request.
