# Supplementary material for: Effects of static exercises on hip muscle fatigue and knee wobble assessed by surface electromyography and inertial measurement unit data
Source: Sci Rep. 2024 May 7;14:10448. doi: 10.1038/s41598-024-61325-7 (PMC11076610; doi:10.1038/s41598-024-61325-7)
Supplement: Supplementary file 1 — Supplementary Information. [file 41598_2024_61325_MOESM1_ESM.docx]

**Supplementary Information**


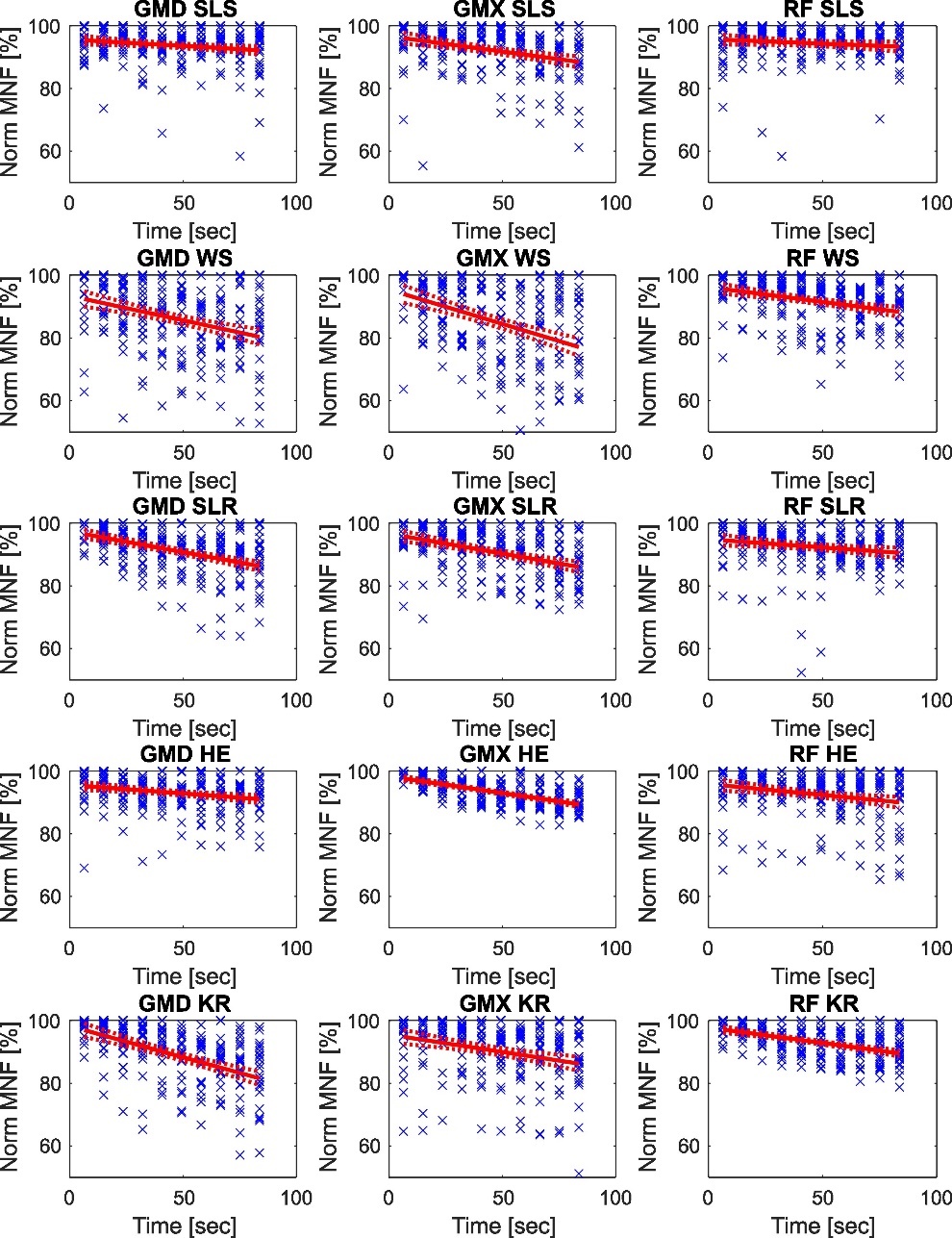


**Figure S1**. Interval data and linear regressions for normalized mean frequency (MNF) broken into individual muscles and individual activities. Data are shown as blue crosses and line of best fit with 90% confidence interval is shown in red. Muscles and activities are denoted as follows: Gluteus medius – GMD, gluteus maximus – GMX, rectus femoris – RF, single leg squat – SLS, wall sit – WS, side leg raise (hip abduction) – SLR, hip extension – HE, and knee raise (hip flexion) – KR.

**Table S1**. Regression slope coefficients and p-values for normalized mean frequency (MNF) broken into individual muscles and individual activities.

| Mean Frequency Regression Results | Gluteus Medius | | Gluteus Maximus | | Rectus Femoris | |
| --- | --- | --- | --- | --- | --- | --- |
|  | Slope [%/sec] | p-value | Slope [%/sec] | p-value | Slope [%/sec] | p-value |
| Single Leg Squat | -0.041 | 0.006 | -0.098 | <0.001 | -0.029 | 0.096 |
| Wall Sit | -0.156 | <0.001 | -0.219 | <0.001 | -0.094 | <0.001 |
| Side Leg Raise | -0.132 | <0.001 | -0.127 | <0.001 | -0.052 | 0.005 |
| Hip Extension | -0.054 | <0.001 | -0.107 | <0.001 | -0.070 | <0.001 |
| Knee Raise | -0.199 | <0.001 | -0.111 | <0.001 | -0.098 | <0.001 |


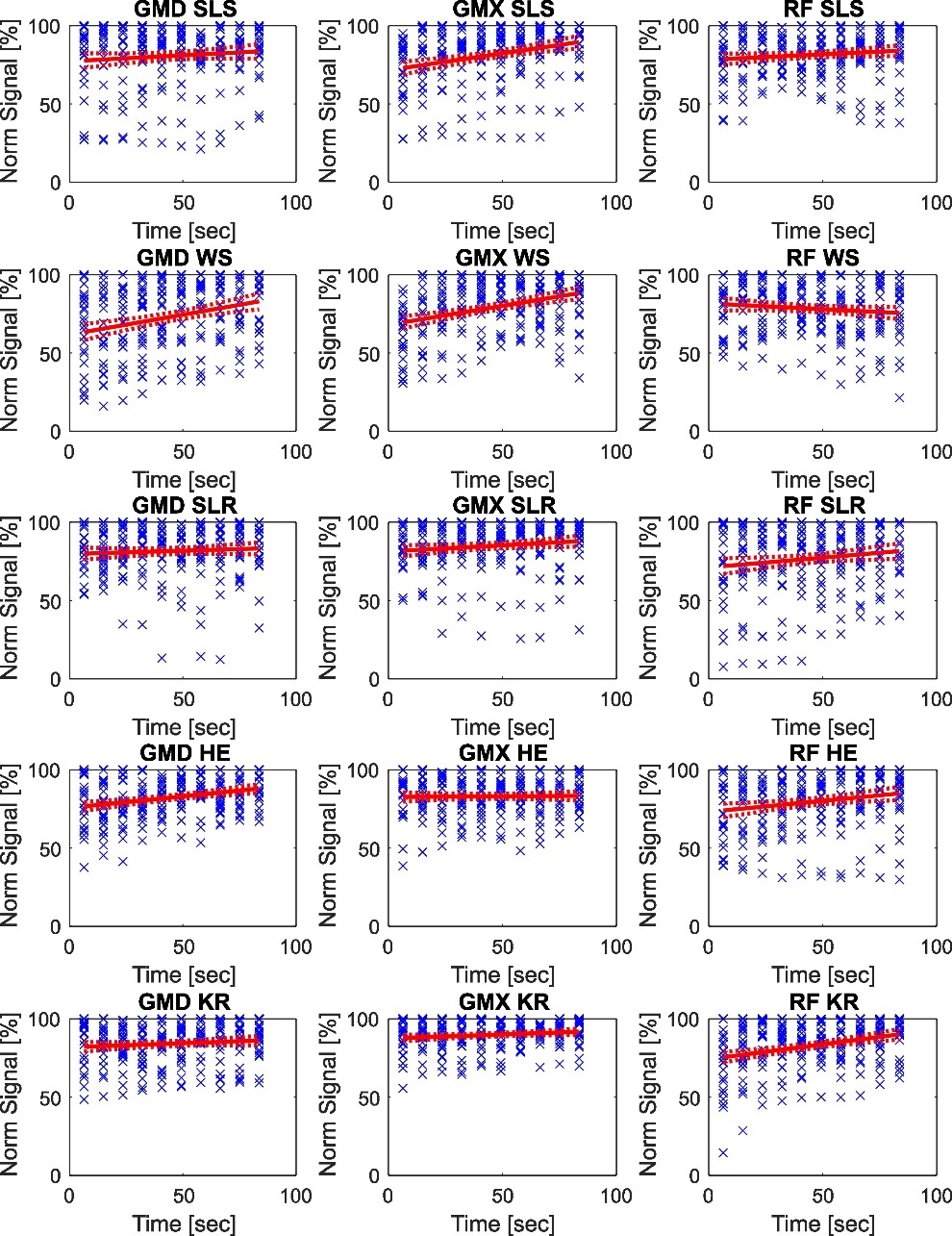


**Figure S2**. Interval data and linear regressions for normalized mean electrical signal broken into individual muscles and individual activities. Data are shown as blue crosses and line of best fit with 90% confidence interval is shown in red. Muscles and activities are denoted as follows: Gluteus medius – GMD, gluteus maximus – GMX, rectus femoris – RF, single leg squat – SLS, wall sit – WS, side leg raise (hip abduction) – SLR, hip extension – HE, and knee raise (hip flexion) – KR.

**Table S2**. Regression slope coefficients and p-values for normalized mean electrical signal broken into individual muscles and individual activities.

| Signal Regression Results | Gluteus Medius | | Gluteus Maximus | | Rectus Femoris | |
| --- | --- | --- | --- | --- | --- | --- |
|  | Slope [%/sec] | p-value | Slope [%/sec] | p-value | Slope [%/sec] | p-value |
| Single Leg Squat | 0.076 | 0.141 | 0.215 | <0.001 | 0.069 | 0.074 |
| Wall Sit | 0.253 | <0.001 | 0.242 | <0.001 | -0.073 | 0.097 |
| Side Leg Raise | 0.042 | 0.320 | 0.078 | 0.047 | 0.125 | 0.025 |
| Hip Extension | 0.148 | <0.001 | 0.009 | 0.795 | 0.141 | 0.003 |
| Knee Raise | 0.052 | 0.120 | 0.051 | 0.026 | 0.190 | <0.001 |


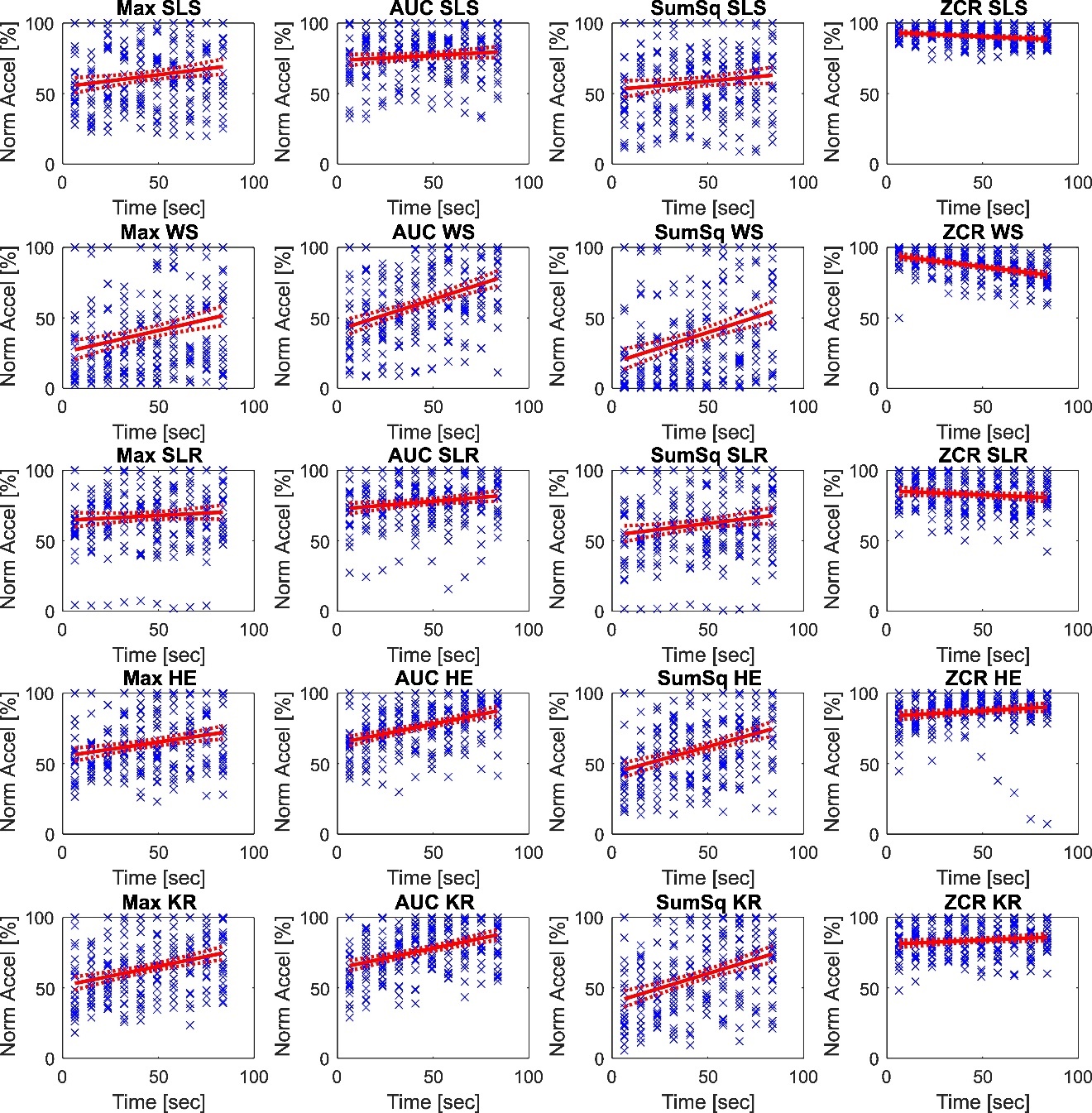


**Figure S3**. Interval data and linear regressions for normalized acceleration measures broken into individual measures and individual activities. Data are shown as blue crosses and line of best fit with 90% confidence interval is shown in red. Measures and activities are denoted as follows: Area maximum absolute acceleration – Max , under absolute acceleration curve – AUC, sum of squared acceleration values – SumSq, zero cross rate – ZCR, single leg squat – SLS, wall sit – WS, side leg raise (hip abduction) – SLR, hip extension – HE, and knee raise (hip flexion) – KR.

**Table S3**. Regression slope coefficients and p-values for normalized acceleration measures broken into individual measures and individual activities.

| Accel Regression Results | Maximum | | Area Under Curve | | Sum of Squares | | Zero Cross Rate | |
| --- | --- | --- | --- | --- | --- | --- | --- | --- |
|  | Slope [%/sec] | p-value | Slope [%/sec] | p-value | Slope [%/sec] | p-value | Slope [%/sec] | p-value |
| Single Leg Squat | 0.170 | 0.005 | 0.072 | 0.102 | 0.124 | 0.053 | -0.058 | <0.001 |
| Wall Sit | 0.312 | <0.001 | 0.438 | <0.001 | 0.436 | <0.001 | -0.170 | <0.001 |
| Side Leg Raise | 0.070 | 0.209 | 0.112 | 0.008 | 0.167 | 0.007 | -0.058 | 0.073 |
| Hip Extension | 0.202 | <0.001 | 0.275 | <0.001 | 0.375 | <0.001 | 0.058 | 0.048 |
| Knee Raise | 0.278 | <0.001 | 0.283 | <0.001 | 0.412 | <0.001 | 0.000 | <0.001 |
